# Impact of Congenital Heart Disease on Brain Development and Neurodevelopmental Outcome

**DOI:** 10.1155/2010/359390

**Published:** 2010-08-24

**Authors:** Mary T. Donofrio, An N. Massaro

**Affiliations:** Children's National Heart Institute, Children's National Medical Center, 111 Michigan Avenue NW, Washington, DC 20010, USA

## Abstract

Advances in cardiac surgical techniques and perioperative intensive care have led to improved survival in babies with congenital heart disease (CHD). While it is true that the majority of children with CHD today will survive, many will have impaired neurodevelopmental outcome across a wide spectrum of domains. While continuing to improve short-term morbidity and mortality is an important goal, recent and ongoing research has focused on defining the impact of CHD on brain development, minimizing postnatal brain injury, and improving long-term outcomes. This paper will review the impact that CHD has on the developing brain of the fetus and infant. Neurologic abnormalities detectable prior to surgery will be described. Potential etiologies of these findings will be discussed, including altered fetal intrauterine growth, cerebral blood flow and brain development, associated congenital brain abnormalities, and risk for postnatal brain injury. Finally, reported neurodevelopmental outcomes after surgical repair of CHD will be reviewed.

## 1. Introduction

Congenital heart disease (CHD) has been reported to occur in 5 to 8 per 1000 live births [1]. It is by far the most common birth defect and a significant cause of childhood morbidity and mortality. Over the past several decades, advances in cardiac surgical techniques and perioperative intensive care have led to improved survival in babies with CHD [1]. While it is true that the majority of children with CHD today will survive, up to half of surviving children will have impaired neurodevelopmental outcome across a wide spectrum of domains [2–5]. While continuing to improve short-term morbidity and mortality is an important goal, recent and ongoing research has focused on defining the impact of CHD on brain development, minimizing postnatal brain injury in this vulnerable population, and improving long-term outcomes for survivors. This paper will review the impact that CHD has on the developing brain of the fetus and infant. Neurologic abnormalities detectable prior to surgery will be described. Potential etiologies of these findings will be discussed, including altered fetal intrauterine growth, cerebral blood flow and neurodevelopment, high prevalence of congenital brain abnormalities, and risk for postnatal brain injury in babies with CHD. Finally, reported neurodevelopmental outcomes after surgical repair of CHD will be reviewed.

## 2. Preoperative Neurological Status in Babies with CHD

Traditionally, studies of neurological outcomes in children with CHD have focused on factors related to surgery, when cerebral perfusion may be compromised during cardiopulmonary bypass. However, the fact that these infants are at risk for adverse outcome before entering the operating room is supported by an increasing body of literature. Brain abnormalities are detectable by preoperative neuroimaging and neurological examination in a significant percentage of infants with CHD. These findings are multifactorial, contributed to by intrauterine hemodynamic alterations, congenital brain abnormalities, and acquired brain injury related to prolonged cyanosis or hypoperfusion after birth. 

### 2.1. Clinical and Radiographic Evidence of Impaired Preoperative Neurological Status

Limperopoulos et al. reported neurobehavioral abnormalities prior to surgery in 56 newborns (<1 month at surgery) and 70 infants (between one month and two years) with complex CHD of a variety of lesion types [6, 7]. In this series, more than 50% of newborns and 38% of infants were found to have abnormalities. In newborns, findings included hypotonia, hypertonia, jitteriness, motor asymmetry, and absent suck. Sixty-two percent had poor behavioral state regulation, 34% feeding difficulties, and 5% seizures. In infants, abnormalities included hypotonia, head preference, lethargy, restlessness, agitation, motor asymmetry, and feeding difficulties. Autistic features were also found. Microcephaly was present in 36%. Newborns with acyanotic lesions were more likely to demonstrate abnormalities than those with cyanotic defects. However, cyanotic infants with an oxygen saturation <85% had a higher incidence of abnormalities. In another report, these authors described preoperative neurological examinations and electroencephalograms (EEG) in 60 infants with CHD. Prior to surgery, 19% of infants had epileptiform activity, and 33% had disturbances in background activity that were moderate or diffuse [8]. EEG abnormalities were associated with abnormal findings on neurological examination, and severe abnormalities were predictive of death. Other authors have likewise reported neurological findings prior to surgery in cohorts of patients with CHD. Chock et al. reported the incidence of an acute neurological event defined as seizure, tone abnormality, or choreoathetosis, to be 19% preoperatively in patients of mixed lesion types [9]. Glauser et al. reported that 38% of infants with hypoplastic left heart syndrome (HLHS) had an abnormal neurological examination or seizures prior to surgery [10]. 

Structural abnormalities and acquired lesions can be detected by neuroimaging performed prior to surgery in a significant percentage of patients with CHD. Preoperative head ultrasounds can detect abnormality in 15–59% of patients with congenital heart disease [9, 11, 12]. Brain magnetic resonance imaging (MRI) performed prior to surgery also demonstrates a high incidence of preoperative brain abnormalities ranging from 25–53% in some series [13–15]. Lesion types detected preoperatively by ultrasound and MRI may be either developmental and/or acquired. In addition to detecting abnormalities, newer imaging modalities may also shed light on pathogenesis. Emerging MR techniques including diffusion tensor imaging (DTI) and advanced MR spectroscopy (MRS) have lead to quantifiable methods to describe brain injury. Miller et al. described results of preoperative DTI and MRS evaluation of 41-term newborns with CHD compared to healthy control newborns without CHD [16]. Decreased ratio of N-acetylaspartate to choline, increased lactate to choline ratio, and decreased white matter fractional anisotropy values were found, similar to profiles measured in preterm infants. This similarity to the preterm brain led the authors to suggest that infants with CHD have abnormal brain development in utero. This concept is further supported by studies of in utero cerebral blood flow in fetuses with CHD.

### 2.2. Intrauterine Factors That Impact Cerebral Flow and Neurodevelopment

The association of complex CHD with intrauterine growth retardation has been well established in numerous reports over the past six decades [17–26]. Biometric data from a regional, population-based, case-controlled study revealed that infants with CHD had abnormal in utero somatic growth compared to matched controls [19]. The study demonstrated that infants with transposition of the great arteries (TGAs) had normal birth weights, but small head circumferences relative to birth weight. Newborns with HLHS had birth weights, lengths, and head circumferences that were less than normal and had head volumes that were disproportionately small relative to birth weight. Finally, infants with tetralogy of Fallot (TOF) had normal proportions, but birth weights, lengths, and head circumferences that were less than normal. There are two theories that have been proposed with regards to the etiology of growth retardation in babies with CHD. First, fetuses with altered growth may have an increased risk of developing cardiac abnormalities [17]. Second, and perhaps more likely, the altered circulation which occurs as a result of the specific structural cardiac abnormality can lead to flow disturbances that may affect in utero growth and brain development [19, 26]. This second theory has been further explored in Doppler ultrasound studies of animal and human fetuses.

#### 2.2.1. In Utero Cerebral Flow Characteristics and Compensatory Mechanisms

**Figure 1:**
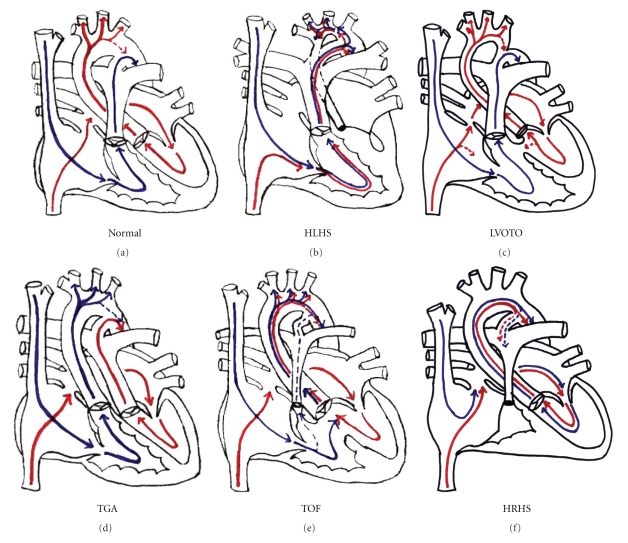
(a) Normal fetal blood flow. (b) Hypoplastic left heart syndrome. (c) left ventricular outflow obstruction. (d) Transposition of the Great Arteries. (e) tetralogy of fallot. (f) hypoplastic right heart. Red arrows: oxygenated blood; blue arrows: deoxygenated blood.

The fetal circulation has been described in detail in both animal and human studies and is depicted in Figure 1(a). Fetal lamb studies have shown that right ventricular output is twice left ventricular output, and oxygen saturation of blood delivered to the cerebral circulation is higher than that delivered to the body through the ductus arteriosus. Deoxygenated blood from the superior vena cava is directed into the right ventricle, across the ductus arteriosus and to the placenta. The Eustachian valve and atrial septum move together to direct deoxygenated blood from the hepatic inferior vena cava into the right ventricle, and oxygenated blood from the ductus venosus across the foramen ovale, through the left ventricle, and to the aorta and cerebral circulation. In the human fetus, the path the blood takes through the heart is identical. Right ventricular output is greater than left, though the difference is not as substantial as in the fetal lamb [27].

In situations where fetal oxygenation is compromised, there is a redistribution of blood flow to the cerebral circulation as a “brain sparing” response [28, 29]. This hemodynamic phenomenon is represented by increased diastolic flow in the cerebral arteries and decreased diastolic flow in the descending aorta and umbilical arteries. Specific regions of the fetal brain may be more protected than others. In a study by Dubiel, interrogation of the middle, anterior, and posterior cerebral arteries was obtained in pregnancies complicated by maternal hypertension and placental dysfunction [29]. Cerebral vasodilation was found in the anterior cerebral artery in 41%, in the posterior cerebral artery in 30%, and in the middle cerebral artery in 24% of fetuses evaluated. Thus, there is an enhanced autoregulatory response of the anterior cerebral arteries, and redistribution of blood flow favors perfusion of the frontal lobes. The middle cerebral arteries, however, have been found to be less reactive and lose reactivity sooner during long-standing compromise. 

Paradoxically, this autoregulatory mechanism has been found to be a harbinger of adverse neurological outcome. Since cerebral vasodilation occurs in the face of compromised fetal oxygenation, the detection of this finding reflects a high risk in utero environment and/or aberrant fetal circulation. In other words, this protective mechanism may be inadequate to maintain normal brain growth and development in situations of prolonged in utero stress. Doppler ultrasound studies have elucidated normative data on blood flow characteristics in human fetuses, and abnormalities have been shown to be predictive of adverse perinatal outcome [30–38]. Resistance and pulsatility indices can both be calculated from Doppler waveform tracings of the cerebral vessels obtained during imaging of the fetus. The resistance index is defined as the systolic flow velocity (SV) minus the diastolic velocity (DV) divided by the SV. The pulsatility index is defined as the SV minus the DV divided by the mean velocity (MV). These indices are considered to be representative of the resistance to flow distal to the point of measurement and can be measured in the cerebral as well as umbilical arterial vessels. Normative information for these indices has been established and lower than normal values have been associated with growth retardation [31, 34, 36, 38] and adverse neurological outcome [33, 36]. A cerebral to umbilical artery ratio of these indices, reflecting degree of “brain sparing” response, is more predictive of intrauterine growth restriction and poor outcome than either index alone. Arbeille showed in normal fetuses that after 15 weeks gestation the cerebral and umbilical resistance both decrease linearly, but the cerebral resistance always remains higher than the umbilical resistance. For the duration of pregnancy the normal cerebral/umbilical resistance ratio is >1.0. In his study, 97% of normal fetuses had cerebral/umbilical ratios >1.0, and 88% of growth-retarded fetuses had ratios <1.0 [38]. In a study by Gramellini, a cerebral/umbilical pulsatility ratio <1.08 after 30 weeks gestation in high-risk pregnancies had a diagnostic accuracy of 70% for growth retardation and a predictive value of 90% for poor perinatal outcome [36]. 

Another ultrasonographic finding indicative of poor outcome is reversal of diastolic flow in the aortic isthmus. In the fetus, the ventricles function in parallel with two distinct shunt pathways: the foramen ovale and ductus arteriosus. These connections equalize pressures in the atria and in the great vessels. Differences between right and left ventricular impedance can be explained by the aortic isthmus which is the narrowest segment of the arch located distal to the left subclavian artery and proximal to ductus arteriosus insertion [27]. Hemodynamically, the fetal aortic isthmus is the bridge between the left ventricular and right ventricular outputs. Normal isthmus blood flow is toward the descending aorta in diastole. Reversed diastolic flow in the isthmus in the absence of CHD is likely due to an altered cerebral/placental resistance ratio caused by placental disease and/or a reflex dilation of the cerebral vasculature in response to hypoxia. Doppler interrogation with reversed flow at the isthmus has been shown to be a predictor of poor neurological outcome in high-risk fetuses [39–41]. 

#### 2.2.2. Alterations in Cerebral Blood Flow in Fetuses with CHD

Several studies have characterized in utero blood flow using Doppler ultrasound in human fetuses with CHD. These ultrasonographic findings and their pattern in CHD infants are summarized in Table 1.

Donofrio et al. published the first multicenter, prospective study that assessed cerebral blood flow by imaging fetuses with CHD at four-week intervals and comparing results to normal controls [42]. Sixty-three studies in 36 heart disease fetuses and 47 studies in 21 normal fetuses were analyzed. Blood flow characterization by lesion type, changes during gestation, and comparisons of indices in CHD versus normal control fetuses were described.

Normal fetal circulation directs oxygenated blood to the brain and deoxygenated blood to the placenta (Figure 1(a)). Fetuses with HLHS likely have increased resistance to cerebral flow as blood flows retrograde across a hypoplastic aortic isthmus to reach the brain. Due to intracardiac mixing, relatively deoxygenated blood supplies the cerebral circulation (Figure 1(b)). Fetuses with left ventricular outflow tract obstruction have varying degrees of resistance to aortic flow, with minimal intracardiac mixing (Figure 1(c)). Fetuses with TGA have venous blood from the cerebral circulation directed back to the brain (Figure 1d). In fetuses with TOF and hypoplastic right heart syndrome relatively deoxygenated blood enters the cerebral circulation due to intracardiac mixing (Figures 1(e) and 1(f)). Thus, lesion type affects not only the source of cerebral blood flow but also the degree of deoxygenated blood distributed through the cerebral circulation.

**Figure 2:**
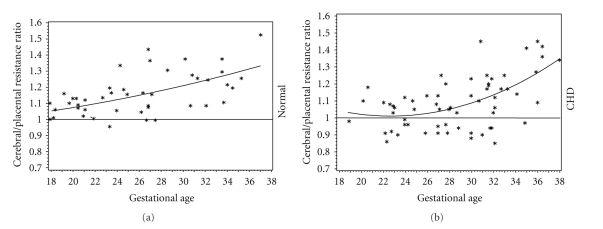
Cerebral to placental resistance ratio versus gestational age for normal fetuses and fetuses with Congenital Heart Disease (CHD). *P is significant normal versus CHD.

Donofrio demonstrated that the cerebral/umbilical resistance ratio versus gestational age relationship was different between normal and heart disease fetuses. Plotting of the data revealed a more quadratic effect in fetuses with heart disease versus the linear relationship in fetuses without CHD. The resistance ratio nadir for heart disease fetuses was at 24-week gestation (Figure 2). This finding is significant since brain development enters a critical period at approximately 24–26-week gestation. Rudolph showed that in normal lamb fetuses, blood flow to the brain begins to increase at a gestational age which correlates to a human age of 26 weeks [27]. Mari showed that cerebral pulsatility decreases in normal fetuses after 24 weeks, indicating increased blood flow to the brain [34]. From 20 to 24 weeks, neuronal proliferation and migration occur, and by 24 weeks the human cerebral cortex has its full complement of neurons. After neuronal migration, the major gyri form between 24–28 weeks gestation with the most rapid increase occurring at about 26 weeks [43]. Thus, autoregulation of cerebral blood flow in fetuses with CHD occurs during a time of brain development when increased perfusion is needed the most to compensate for cerebral hypoxemia.

Overall, mean cerebral artery resistance and mean cerebral/umbilical resistance ratios were lower for fetuses with heart disease compared to normal. When comparing individual groups, mean cerebral/umbilical resistance ratios were lower for fetuses with HLHS compared to normal. Fetuses with TGA had the second lowest mean ratios, with a trend towards significance. The percent of fetuses in each group with at least one abnormal cerebral/umbilical resistance ratio during gestation was different when comparing normal and heart disease groups (5% versus 44%, resp.). In this analysis, fetuses with hypoplastic left and right heart syndrome had the highest incidence of abnormal cerebral/umbilical resistance ratios (58% in HLHS and 60% in hypoplastic right heart syndrome). Fetuses with TOF and TGA were less affected (45% and 25%, resp.), and no fetus with left ventricular outflow tract obstruction had an abnormal resistance ratio. Mean head circumference to fetal weight ratio trended towards being smaller when comparing normal and heart disease fetuses. Abnormal cerebral/umbilical resistance ratios at fetal weights around 2 kg were associated with smaller head circumferences in CHD fetuses.

The second study to evaluate cerebral blood flow in fetus with CHD was a cross-sectional, prospective analysis of fetuses with HLHS (*n* = 28), and defects with left (*n* = 13) or right (*n* = 17) heart obstruction compared to 114 normal controls published by Kaltman [44]. Cerebral (CPI) and umbilical pulsatility indices (UPI) were measured, and comparisons were made using Z-scores generated from published normative data. When comparing groups, the HLHS group had a lower mean CPI while the right sided obstruction group had a higher CPI than normal. The left-sided obstruction group was not different from normal. The right-sided obstructive lesions had a higher mean UPI while both other heart disease groups were not different from normal. Of note, the UPI/CPI ratio was not different when comparing groups. The finding of increased CPI in fetuses with right sided obstructed flow was different from the results of Donofrio's study. It is possible that cerebral resistance is increased in these defects because antegrade flow from the aorta is unobstructed and perhaps increased from normal. The increase in cerebral resistance may be due to cerebral autoregulation to limit excessive flow. The difference between the findings of the two studies may be in part attributable to the fact that in Kaltman's study, fetuses with right sided obstruction included those with TOF which were analyzed separately by Donofrio. Kaltman's fetuses had an increased umbilical resistance that was also not found in Donofrio's study. In a study by Meise [45] umbilical resistance was abnormal in some fetuses with heart disease. The true prevalence of altered placental flow in fetuses with CHD is not known. Of note, in Meise's study no difference was found in cerebral resistance when comparing normal to CHD fetuses; however defects were not separated into physiologic subtypes for analysis. 

Other studies have revealed similar findings. In a study by Jouannic [46] cerebral flow in fetuses with TGA was evaluated. CPI in TGA fetuses (*n* = 23) was compared to 40 normal matched controls. In this study the CPI was significantly lower in TGA fetuses compared to normal fetuses. Umbilical Doppler (including UPI) and ductus venosus flow were normal. This study confirmed the trend seen in Donofrio's study and suggests that fetuses with TGA have cerebral vasodilation likely from cerebral hypoxia related to the structural cardiac abnormality. In a study by Modena's [47], CPI in 71 fetuses with CHD was compared to matched controls. Cardiac lesions were grouped into defects with intracardiac mixing of deoxygenated and oxygenated blood versus those that were considered to have nonmixing lesions. Abnormal CPI was found in 7% of fetuses with CHD, versus none with normal hearts. All abnormal CPI occurred in fetuses with intracardiac mixing suggesting that hypoxemia plays a role in cerebral vasodilation. Guorong [48] evaluated 45 fetuses with CHD and compared findings to 275 controls. CPI, UPI, and UPI/CPI ratios were calculated and converted to z-scores similar to the study of Kaltman. They found that fetuses with CHD had normal CPI; however UPI/CPI ratios were elevated suggesting a redistribution of the circulation towards the head. Fetuses with CHD complicated by congestive heart failure had low CPI compared to normal suggesting cerebral vasodilation. They concluded that cerebral dilation occurs as a result of cerebral hypoxemia from limited perfusion and that it is both heart function and type of CHD that have an impact on fetal cerebral blood flow distribution and postnatal neurologic outcome.

The results of these studies all demonstrate that alterations in the intracardiac circulation caused by specific cardiac defects result in changes in cerebral blood flow characteristics that can be documented by Doppler ultrasound. The mechanism is complex and likely is related to both the cerebral oxygen content of blood and also the oxygen delivery which is dependent on cardiac function and total combined cardiac output. In Donofrio's study, hypoplastic left and right heart fetuses were the most affected. These defects both have a single ventricular chamber with intracardiac mixing of blood and thus lower oxygen content in the blood that is delivered to the brain. This relative cerebral hypoxemia may stimulate a decrease in cerebral vascular resistance that in the presence of an unchanged placental resistance results in cerebral vasodilation and abnormal cerebral/umbilical resistance ratios. It has been shown that the fetal myocardium delivers less active tension, generates a lower maximum force of contraction, and responds less to increased preload than mature myocardium [49]. The single ventricular chamber may not be able to increase combined ventricular output enough to compensate for the cerebral hypoxemia caused by intracardiac mixing of blood. This may lead to abnormal brain development despite cerebral autoregulation. In HLHS, cerebral perfusion is likely also limited by the increased resistance caused by the hypoplastic aortic isthmus. Since the cerebral circulation is supplied retrogradely, the isthmus may restrict the amount of blood that can be delivered to the brain, despite the protective autoregulatory mechanism of the cerebral vascular bed. This may contribute to the higher incidence of neurodevelopmental abnormalities found in these children. In contrast, fetuses with TGA and TOF were less affected than those with single ventricles. In these defects, there is intracardiac mixing of blood in the presence of two ventricles with no obstruction to antegrade cerebral flow. These hearts may be able to compensate for the cerebral hypoxemia by increasing combined ventricular output. Of note, fetuses with left ventricular outflow tract obstruction were not affected. These fetuses likely have cerebral blood with near normal oxygen content and adequate antegrade aortic flow to support cerebral perfusion. 

Ultimately, what is of utmost importance is how much oxygen and substrate actually reache the brain in fetuses with CHD. Chock et al. reported that elevated nucleated red blood cell counts measured after birth (presumably a marker of the erythropoietic response to chronic in utero hypoxia) was a significant risk factor (odds ratio 7, *P* = .02) for a perioperative acute neurological event [9]. Multiple factors including cardiac output, oxygen content, and hemoglobin content are all critical factors that will impact on the oxygen and substrate delivery to the brain, in addition to the relative resistances of the distal vascular beds. Given the findings of neurological abnormalities after birth and before surgery in these infants (including smaller head circumference, clinical neurological abnormalities, and radiographic abnormalities), it appears that there is inadequate fetal cerebral oxygen delivery to support normal brain development even in the presence of the autoregulatory “brain sparing” response. Further studies are needed to correlate these alterations in fetal circulatory dynamics with postnatal neurodevelopmental outcome and to see if intervening in cases where significant abnormalities are detected may improve outcome for this population.

### 2.3. Congenital Brain and Developmental Abnormalities

Structural and functional brain abnormalities have been identified in babies born with CHD in the neonatal period prior to surgical repair. In addition, there have been studies showing that similar abnormalities can be found in fetuses. These brain abnormalities represent a spectrum of disease processes with multiple potential etiologies. Abnormalities indicative of altered development have been noted in addition to abnormalities relating to injury from embolic events or hypoxia. In addition, genetic abnormalities have been incited in both the cause of structural brain defects as well as leading to fragility of the brain which may increase the potential for injury. 

#### 2.3.1. Genetic Abnormalities Associated with CHD

Genetic syndromes that have associated neurological abnormalities are present in many children with CHD [50]. For example, infants with Trisomy 21, DiGeorge, velocardiofacial (VCFS), Turner's, and William's syndromes have common congenital heart abnormalities and varying degrees of associated developmental delay. The incidence of CHD in patients with Trisomy 21 is approximately 40%, with the most common being defects of the endocardial cushion and ventricular septum. All infants with Trisomy 21 have mental retardation, ranging from mild to severe. DiGeorge syndrome and velocardiofacial syndrome, both caused by a microdeletion on chromosome 22 and associated with developmental delay, are also associated with conotruncal defects including interrupted aortic arch and truncus arteriosus. Turner's syndrome (due to absence of an X chromosome) is associated with mildly decreased intelligence quotient (IQ) scores and abnormalities of the aortic valve and coarctation. Finally, in William's syndrome, which is due to a chromosome mutation on 7q11 and associated with cognitive and behavioral abnormalities, common cardiac defects include supravalvar aortic stenosis and peripheral pulmonary branch stenosis. Other genetic factors may also place certain patients at higher risk for CNS injury. There is increasing evidence that Apolipoprotein E (APOE) is important for neuronal repair. APOE e2 allele carriers were found to have significantly lower Bayley PDI at one year of age after cardiac surgery compared to those without the allele, suggesting a genetic susceptibility to brain injury [51]. 

#### 2.3.2. Clinical Presentation of Preoperative Brain Abnormalities

Abnormalities on neurological exam have been detected preoperatively in neonates with CHD. Chock reported an incidence of an acute neurological event defined as seizure, tone abnormality, or choreoathetosis, to be 17% in patients with CHD prior to surgery [9]. In a study of infants with HLHS by Glaucer, 38% were reported to have an abnormal neurological exam or seizures prior to surgery [10]. A prospective study of babies with CHD by Limperopolous revealed preoperative neurological abnormalities in 50% of newborns and 38% of infants [6]. Abnormalities included hypotonia, hypertonia, jitteriness, motor asymmetry, and absent suck. Sixty-two percent had poor behavioral state of consciousness regulation, 34% feeding difficulties, and 5% seizures. In infants, abnormalities included hypotonia, head preference, lethargy, restlessness and agitation, motor asymmetry, and feeding difficulties. Autistic features were found. Abnormalities were independent of hemodynamic instability. In another study by Limperoupoulos, 19% of infants had epileptiform activity, and 33% had disturbances in background activity on electroencephalogram (EEG) that were moderate or diffuse [8]. 

#### 2.3.3. Structural Brain Abnormalities in Newborns with CHD

It is well described that there is a high incidence of brain malformation in babies with CHD in the absence of a defined genetic syndrome [52, 53]. These brain findings may be due to undefined genetic abnormalities or may relate to alterations in the circulation leading to injury or abnormal or delayed brain development. In one autopsy study, multiple congenital brain anomalies were found in a significant proportion of babies with HLHS [53], including marked microcephaly (brain weight > 2 standard deviations below the normal mean) in 27%, abnormal cortical mantle formation in 27%, and overt central nervous system malformations such as agenesis of the corpus callosum or holoprosencephaly in 10%. The absence of dysmorphic features did not preclude the presence of central nervous system malformations, and conversely, the presence of dysmorphic features did not reliably predict the presence of a brain abnormality in this study. These structural abnormalities have been detected in living patients by physical exam and neuroimaging performed prior to surgery. In a study by Limperopoulos, preoperative evaluation of babies with CHD excluding HLHS revealed microcephaly in 36% and macrocephaly in 13% of babies evaluated [7]. Head ultrasound has detected abnormality in patients with CHD including cerebral atrophy [11], echodensities or calcifications in the basal ganglia [11, 12], and widened ventricular or subarachnoid spaces [9, 12, 52, 53]. 

Brain MRI performed prior to surgery has also demonstrated a high incidence of preoperative brain abnormalities. In a study by Mahle, 24 patients with CHD were studied, and the only brain anomaly believed to be congenital in origin was an open operculum which was present in 17% [14]. In a study by Licht in 25 babies with CHD, 53% with CHD had developmental and/or acquired brain lesions including microcephaly (24%), incomplete closure of the operculum (16%), and PVL (28%) [15]. Miller, using MRS and diffusion tensor imaging (DTI) in neonates with CHD, found abnormalities similar to the preterm population [16]. Utilizing biochemical ratios, a higher ratio of lactate/choline and a lower ratio of N-acetylaspartate/choline were found in CHD patients with TGA or single ventricle physiology compared to controls. N-acetylasparatate is a marker of neuronal integrity found in high concentrations in neurons and is known to increase with maturation. In contrast, lactate decreases with increasing maturity. These results suggest findings of an immature brain in full-term neonates with CHD. In the same cohort it was also noted that average diffusivity was increased in the CHD neonates. Average diffusivity decreases with development; this is thought to be due to decreased water content and increased membrane growth in neuronal and glial cells that accompany brain maturation. In a study by Licht [54], infants with HLHS and TGA were evaluated by MRI with outcome measures being head circumference and total brain maturation score. The brain maturation score is a published scoring system that evaluates four parameters that include myelination, cortical infolding, involution of glial cell migration bands, and the presence of germinal matrix tissue. In this study, the mean head circumference was one standard deviation below normal and the mean total maturation score for the cohort was significantly lower than reported normative data suggesting a delay in maturation of one month for babies with CHD. Limperopoulos evaluated 55 fetuses with CHD using MRI and MRS and compared results to 50 normal fetuses [55]. Fetal intracranial cavity volume, cerebrospinal fluid volume, and total brain volume were calculated using 3-dimensinal volumetric MRI, and cerebral N-acetyl aspartate/choline ratios and cerebral lactate levels were determined using MRS. Gestational age at study ranged from 25 to 37 weeks. MRI analysis showed a progressive decline in age-adjusted total brain volume and intracranial cavity volume in fetuses with CHD relative to controls. N-acetyl aspartate/choline ratios increased in CHD fetuses but were slower to rise than what was seen in normal fetuses. On multivariable analysis, the cardiac diagnosis and percentage of combined ventricular output through the aortic valve were independently associated with total brain volume. Predictors of lower acetyl N-acetylaspartate/choline ratios included cardiac diagnosis, absence of antegrade aortic arch flow, and evidence of cerebral lactate. These results suggest that in the third trimester fetuses with some forms of CHD, in particular those with diminished aortic output, have evidence of impaired development and brain metabolism and have total brain volumes that are lower than normal. These findings noted on fetal MRI by Limperopoulos, and on neonatal preoperative MRI by Miller and Licht, suggest that the altered circulation that attends the fetal circulation in specific CHD may delay brain maturation and growth and play a role in the neurodevelopmental impairments of these patients.

### 2.4. Acquired Preoperative Brain Injury

In addition to the developmental lesions previously mentioned, acquired lesions may be detectable in babies with CHD before surgery representing injury due to hemodynamic compromise with or without hypoxia. There have been several studies that have revealed the presence of hypoxic ischemic injury before surgical intervention. In an autopsy study, Glauser found that 45% of babies with HLHS, half of whom did not undergo surgery, had hypoxic ischemic lesions and/or intracranial hemorrhage [10]. Hypoxic ischemic injury included cerebral necrosis, periventricular leukomalacia (PVL), and brainstem necrosis. Lesions detected by cranial ultrasound studies include intraventricular hemorrhage [9], cerebral atrophy [11], echodensities or calcifications in the basal ganglia [11, 12], widened ventricular or subarachnoid spaces [9, 12, 52, 53], and ischemic changes [10, 12]. Licht found PVL in 28% of CHD patients imaged with MRI preoperatively [15]. In Mahle's study, 25% of patients had clear evidence of ischemic injury including PVL or infarct on brain MRI done before surgery. Over 50% of patients had elevated lactate on MR spectroscopy, further evidence of brain ischemia [14]. In one of the most recent and largest cohort of 62 patients studied prospectively with MRI, McQuillen described radiographic evidence of brain injury in 39% of patients, most commonly stroke followed by white matter injury [13]. The high incidence of white matter injury, particularly PVL, is notable in this population and atypical of the pattern in term newborns who suffer hypoxia-ischemia from other causes. This finding suggests that some of the acquired brain injuries in babies with CHD may be related to an abnormality of the cerebral vascular bed and/or brain development. It is possible that babies with CHD have white matter cell lines that are particularly susceptible to injury, similar to the vulnerability of these cells in preterm infants, manifesting in different patterns of injury compared to other term infants who suffer hypoxic ischemic injury.

In addition to neuroimaging evidence of preoperative brain injury, various serum biomarkers reflecting systemic and neurological compromise have been examined in patients with CHD. Serum lactate as a marker of global hypoperfusion has been followed and found to be elevated preoperatively in patients with CHD [56]. Proinflammatory cytokine profiles (with elevated IL-6 and decreased IL-10) have been detected in infants with cardiac disease preoperatively [57]. The glial-derived protein S100B has been evaluated in infants and adults with cardiac disease and is believed to be a marker for cerebral ischemia [58–60]. S100B levels have been found to be elevated in infants with CHD before surgery, possibly reflecting preexisting neurological injury [60]. Infants with HLHS had the highest levels before surgery, and the S100B concentration correlated inversely with the size of the ascending aorta. This finding complements the Doppler studies that suggest that the amount of antegrade flow in the aorta in fetuses and infants with HLHS impacts cerebral perfusion and may leave these infants particularly prone to ischemic injury even before surgery. 

## 3. Neurodevelopmental Outcome after Surgical Repair of CHD

There is an increasing body of literature reporting intermediate and long-term outcomes from various cohorts of patients with CHD. Generalizations are problematic since there is heterogeneity amongst these reports in regards to surgical/medical era, patient population, type of cardiac lesion, age at followup, and type of assessment tool used. Given the advances in care including improved medical and surgical techniques and brain protection strategies, the more historical data reported may not reflect the improved outcomes that we hope to see with patients in the current era. Nevertheless, outcome data has provided some general knowledge about these patients postoperatively, both in the short-term and for long term survivors.

### 3.1. Short-Term Neurological Outcome after Surgery

Postoperative short-term outcomes include a significant incidence of neurological abnormalities. Miller performed postoperative neurological examinations in 91 infants who had undergone heart surgery and found 15% had clinical seizures, 34% had hypotonia, 7% had hypertonia, 5% had asymmetry of tone, and 19% had decreased alertness at hospital discharge [61]. The aforementioned study by Limperopoulos that detected high incidence of preoperative neurological findings also reported that these abnormalities generally persisted or worsened postoperatively, with additional findings of cranial nerve abnormalities and choreoathetosis [6]. Chock reported the incidence of an acute neurological event to be 25% within the first week after surgery and 56% after the first week [9]. In other reports, the incidence of clinical post-operative seizure was 4–11% and was detected by continuous EEG monitoring in up to 20% of patients in the 48 hours following surgery [62–67]. Swallow dysfunction [68] or abnormal suck [6] is another significant finding in patients with CHD following surgery, as feeding performance has been shown to be an early indicator of later neurodevelopmental outcome [69].

### 3.2. Neurodevelopmental Outcomes for Survivors at School Age and Beyond

Reports of long-term followup have limited applicability as technological advances in medical management and surgical repair techniques make outcomes reported from procedures done in previous decades less insightful. For example, of note when interpreting results of the outcomes from the Boston Circulatory Arrest Trial is that during the time period of the study the alpha stat strategy, hemodilution to 20% hematocrit, and currently outmoded hardware for bypass were utilized. Thus, consideration must be made to surgical era when interpreting results from historical cohorts. Despite this, some general themes have arisen from reported long-term outcomes in patients with CHD.

Long-term survivors, although they frequently have limitations, are actually quite functional. Several studies have demonstrated that while patients with CHD have significantly lower mean IQs than age-matched controls, their IQs still fall within the normal range [4, 5, 70–94]. These reports, however, also describe highly prevalent gross and fine motor, attention, and school problems. The prospective cohort originally described by Limperoulous was followed through school age by Majnemer and found to have high incidence of gross and fine motor abnormalities (49 and 39%, resp.) and abnormal neurological examination (28%) [2]. Highly prevalent behavioral and school problems are reported from the Boston cohort [5, 91]. Overall long-term cognitive outcomes reported from various studies are presented in Table 2. Special attention should be paid to outcomes reported for two specific populations: those with single ventricles and TGA. Patients with single ventricles, especially those with HLHS, have higher risk for adverse outcome based on their underlying physiology and hemodynamics as well as complexity of surgical repair. Meanwhile, patients with TGA have been the most extensively and reliably studied.

### 3.3. Neurodevelopmental Outcomes in Children with Single Ventricles

Children with a single functioning ventricle who undergo a series of palliative surgical procedures culminating in the Fontan operation are at the highest risk of developmental compromise. In addition to the frequent hemodynamic instability accompanying the complex cardiac physiology in these patients, surgical repair often involves multiple operations requiring bypass and circulatory arrest. Children with HLHS are at particular risk for neurodevelopmental abnormalities, in part because the repair involves a period of hypothermic circulatory arrest that in the past has been longer than 30–40 minutes. There have been several reports of neurodevelopmental outcomes in these patients although none involved prospectively identified cohorts. Forbess reported outcomes from the registry of outcome data maintained by the group at Boston Children's Hospital. He reported that patients with single ventricle lesions had significantly lower full-scale and performance IQ and had lower scores on multiple domains of memory, learning and visual-motor testing, when compared to patients undergoing biventricular repair [87]. Wernovsky evaluated 133 patients who had a Fontan operation between the years 1973–91 [71]. For 128 patients who underwent cognitive testing, the mean full scale IQ of 96 was lower than the population mean. Mental retardation was found in 8%. Children with HLHS scored lower on all parameters compared to children with other single ventricle lesions. Mahle reported results of outcomes for patients with HLHS who underwent surgery in 1984–1991 [78]. In 28 children, the median full scale IQ was 86, lower than the general population mean. Performance IQ was lower than verbal (83 versus 90). Full scale IQ scores in the mental retardation range were found in 18%. Cerebral palsy with hemiparesis was documented in 17%, microcephaly in 13%, fine motor abnormalities in 48%, gross motor abnormalities in 39%, and speech deficits in 30%. Goldberg reported IQ scores reflecting outcomes for HLHS in the time period between 1989–94 [85]. Children with HLHS fell within the normal range for full scale IQ as measured by the Weschler test but scored lower than children with other single ventricle lesions. For the whole group, IQ was 101, for HLHS 94, and for other single ventricles 107. Brosig reported the most recent outcome data for patients who underwent surgery between 1993–99 [90]. Median IQ was 97 in 13 patients evaluated at 3.5 to 6 years. When compared to patients with TGA, patients with HLHS had more problems with visual-motor skills, expressive language, attention, and externalizing behavior.

### 3.4. Neurodevelopmental Outcomes in Children with TGA

The majority of neurodevelopmental outcome reports come from cohorts of patients retrospectively identified and recalled for developmental assessments, leading to inherent selection bias. There are few prospective studies which have provided more reliable outcome information. The Boston Circulatory Arrest Trial evaluated differences between two groups of babies with TGA based on bypass strategy and provided insight into the outcomes of babies with TGA in general after surgical repair [5, 91–93]. The overall study population had significantly lower mean IQ than the general population although still within the normal range. Verbal IQ was higher than performance IQ. Although mean scores on most outcomes were also within normal limits, the cohort as a whole was performing below expectations in several domains including academic achievement, gross and fine motor, working memory, attention, and higher-order language skills. There was high prevalence of hearing deficits (12% and 8% in the circulatory arrest and low flow bypass groups, resp., *P* = .43) at four years [91] and abnormalities on neurological exam (circulatory arrest 71% and low flow bypass 64%, *P* = .23) at eight years [5]. At 8 years following repair, 30% of the population were receiving remedial services in school, and 10% had repeated a grade.

In another longitudinal prospective study in Germany, Hovels-Gurich et al. followed 60 patients with TGA through school age and have reported outcomes for their group at a mean of 10 years [4, 82, 94]. While they found that overall IQ scores were not significantly different than population norms (different from the Boston cohort), they reported high rates of gross and fine motor dysfunction (>20%), reduced expressive and receptive language (around 20%), and speech abnormalities (40%).

## 4. Conclusion

Advances in medicine, including prenatal diagnosis and evaluation, innovations in cardiothoracic surgical techniques, and improvements in perioperative management have contributed to the increased survival of infants with CHD. Greater attention is now being directed toward understanding how in utero hemodynamics affect cerebral development, how the conduct of the operation can best be manipulated to maximize cerebral oxygen delivery and utilization, and how perioperative care, including the incorporation of neuromonitoring, can be optimized. Although as a group IQ appears to be in the normal range for CHD survivors, rates of neurodevelopmental impairment continue to be significant. Neurodevelopmental evaluation in patients with CHD should be standard practice to not only identify those with impairments who would benefit from intervention services but also to continue to identify risk factors and strategies to optimize both short- and long-term outcomes in these high-risk children. In the future, fetal management and intervention strategies for specific defects may ultimately play a role to improve in utero hemodynamics and increase cerebral oxygen delivery to enhance brain growth and improve early neurodevelopment.

## Figures and Tables

**Table 1:** Doppler indices of cerebral blood flow.

Doppler Ultrasound Finding	Definition	Significance	Congenital Heart Disease
Cerebral Pulsatility Index (CPI)	(SV-DV)/MV	Lower value associated with higher mortality, growth retardation, and poor neurological outcome	Lower in HLHS and higher in right-sided obstruction lesions compared to normal [44] Lower in TGA [46] Lower in CHD with intracardiac mixing [47] Lower in CHD fetuses with CHF [48]

Cerebral Resistance Index (CRI)	(SV-DV)/SV	Lower value associated with growth retardation	Lower in CHD infants compared to normal [42]

Cerebral/Umbilical Pulsatility Ratio	CPI/UPI	Ratio <1 associated with growth retardation and poor perinatal outcome	No difference [44] Increased in CHD [48]

Cerebral/Umbilical Resistance Ratio	CRI/URI	Ratio <1 associated with growth retardation	Lower in CHD infants compared to normal Lowest in HLHS (58% with ratio <1) [42]

Reversal of diastolic flow in the aortic isthmus	Blood flow away from the descending aorta	Predicts poor neurological outcome	Present in HLHS and severe left ventricular (LV) outflow obstruction with LV failure

**Table 2:** Reported long-term cognitive outcomes for patients with congenital heart disease.

Diagnosis	Reference	Number of Patients	Mean age at assessment (range)	Cohort prospectively identified	Surgical era	Full scale IQ (mean ± SD) or median (range)
CHD-mixed	Clarkson, 1980 [89]	72	4y (2.4–7y)	No	1969–71	93 ± 16
	Forbess, 2002 [87]	243	5 y	No	1993–2000	97 ± 16
	Miatton, 2007 [75]	43	8y	No	1995–99	96 ± 15
TGA	Clarkson, 1980 [89]	22	4y (2.4–7y)	No	1969–71	90 ± 18
	Newberger, 1984 [74]	33	5.8y (5.5–6.3y)	No	1968–72	102 ± 15
	Hesz, 1988 [84]	10	(6.5–14y)	No	1967–84	92 ± 12
	Oates, 1995 [73]	30	(9–10y)	No	1972–82	100 ± 17
	Ellerbeck, 1998 [88]	54	8y	No	1981–90	90 ± 21
	Hovels-Gurich, 1997 [82] 2001 [94]	77 60	5 y (3–9y) 10y (8–14y)	Yes	1986–92	99 ± 14 99 ± 17
	Bellinger, 1999 [91] 2003 [5]	158 155	4y 8y (7–10y)	Yes (Boston CA)	1988–92	93+17 97+15
	Karl, 2004 [81]	74	9y (4–14y)	No	1988–94	102 ± 13
	Brosig, 2007 [90]	13	(3.5–6y)	No	1996–99	110 (90-126)
	Clarkson, 1980 [89]	17	4y (2.4–7y)	No	1969–71	88 ± 14
TOF	Oates, 1995 [73]	51	(9–10y)	No	1972–82	100 ± 17
	Hovels-Gurich, 2006 [83]	20	7y (5–12y)	Yes	1993–99	91 ± 13
	Miatton, 2007 [76]	18	8y	No	1994–99	95 ± 14
	Clarkson, 1980 [89]	16	4y (2.4–7y)	No	1969–71	93 ± 15
TGA/TOF	Wright, 1994 [70]	29	9.5y (7–12y)	No	1979–84	94 ± 15
VSD	Oates, 1995 [73]	33	(9–10y)	No	1972–82	102 ± 12
	Hovels-Gurich, 2006 [83]	20	7y (5–12y)	Yes	1993–99	93 ± 12
SV	Wernovsky, 2000 [71]	128	11y (3.7-41y)	No	1973–91	96 ± 17
	Uzark, 1998 [72]	32	2.5y (1.5–9.5y)	No	1986–94	98 ± 12
	Goldberg, 2000 [85]	51	5y (2.8–8y)	No	1989–96	101 ± 5
	Forbess, 2002 [87]	34	5 y	No	1993–2000	90 ± 16
HLHS	Kern, 1998 [80]	12	4y (3–6y)	No	1990–96	80 ± 14
	Goldberg, 2000 [85]	26	5y (2.8–8y)	No	1989–96	94 ± 7
	Mahle, 2000 [78]	28	9y (6–13.6y)	No	1984–91	86 (50–116)
	Mahle, 2006 [77]	48	12y (8–17y)	No	1986–94	86 ± 14
	Brosig, 2007 [90]	13	(3.5–6y)	No	1996–99	97 (71–112)
